# Supplementary material for: Cell-Type Apoptosis in Lung during SARS-CoV-2 Infection
Source: Pathogens. 2021 Apr 23;10(5):509. doi: 10.3390/pathogens10050509 (PMC8145065; doi:10.3390/pathogens10050509)
Supplement: Supplementary file 1 [file pathogens-10-00509-s001.zip › pathogens-1159551-supplementary.pdf]

Supplementary materials

Cell culture

HUVECs (Cell Applications, Atlanta, GA) were cultured in Endothelial Cell Growth Medium (Cell Applications). Vero (E6) cells and BEAS2B cells were cultured in Dulbecco's modified Eagle's medium (DMEM, Corning) supplemented with 10% fetal bovine sera.

DNA extraction from formalin-fixed NHP lung tissues and DNA ladder assays

DNA samples were extracted from formalin-fixed NHP lung tissues using the DNeasy Blood & Tissue kit (Qiagen, Germantown, MD), following manufacturer's instruction, in particular for formalin-fixed tissue samples. For the DNA ladder assays, either 2 μg or 6 μg DNA samples were loaded into a 1% agarose gel prior to electrophoresis for 1 hour at 50V. Whole cell lyses of H_2O_2 (500 μM, 48 hours)-treated Vero cells were used as a positive control. Gels were imaged using the Bio-Rad DNA gel imaging system (Bio-Rad Universal Hood II Gel Doc System).

Antibodies and other reagents

Anti-caveolin-1 rabbit antibody was purchased from Novus Biologicals (Centennial, CO). Anti-CD3 rabbit antibody was purchased from DAKO (Glostrup, DK). Anti-CD31, anti-surfactant protein C (SPC), anti-CD68, anti-cytochrome C, anti-FasL, anti-caspase-9, anti-PARP and anti-SARS-CoV-2 rabbit antibodies were purchased from Abclonal (Woburn, MA). AlexaFluor 594-conjugated goat anti-rabbit IgG and DAPI were

purchased from Invitrogen (Carlsbad, CA). Normal mouse and rabbit IgGs were purchased from Agilent (Santa Clara, CA). EPAC1-specific agonist (ESA) I942 was was provided by Dr. Zhou. Fetal bovine sera were obtained from Cell Applications. DMEM medium was purchased from Corning (Corning, NY). In situ cell death detection kits (TUNEL fluorescein) were purchased from Sigma (Louis, MO). Pierce™BCA protein assay kits were purchased from Thermo Fisher Scientific (Rockford, IL). Unless otherwise indicated, all reagents were purchased from Thermo Fisher Scientific.

Immunofluorescence (IF) staining and hematoxylin and eosin (H&E) staining

Tissues were fixed in 4% neutral buffered formaldehyde, embedded in paraffin, sectioned at 5 μm thickness, and processed by H&E for basic histological assessment following the published method for NHP model of SARS-CoV-2 infection[1, 2]. For IF study, after antigen retrieval and blocking, lung sections were incubated with CD31, caveolin-1, SPC, CD68, CD3, cytochrome C, FasL, or SARS-CoV-2 antibody (all at 1:500) for 2 hours prior to incubation with Alexa Fluor 594-conjugated goat anti-rabbit IgG (1:1000) for 1 hour. A rabbit polyclonal IgG (Thermo Fisher) served as a negative control. Nuclei were stained with DAPI. Fluorescent images were reviewed using an Olympus BX51-microscope prior to further analysis using a Nikon A1R MP ECLIPSE T*i* confocal microscope.

Confocal microscopy [3]

Fluorescent staining was viewed and images were captured using a Nikon A1R MP ECLIPSE T*i* confocal microscope equipped with *NIS*-Elements imaging software version

4.50.00 (Nikon, Tokyo, Japan). Filters were selected to avoid crosstalk between channels. Alexa 594 was excited with a 561 nm laser. The DAPI signal was excited with a 405 nm laser.

Western blotting [4]

For western blotting, equal amounts of soluble proteins were subjected to 10% SDS–polyacrylamide gel electrophoresis (SDS-PAGE). Proteins were transferred onto a polyvinylidene difluoride membrane and then incubated with primary antibody (1:1,000 for anti-PARP, SARS-CoV-2, caspase-9, or GAPDH antibodies) at 4°C overnight, followed by incubation with a secondary antibody at 1:10,000 for 2 hours. A goat anti-mouse or rabbit IgG and IgM (H+L)-HRP (Thermo Fisher Scientific) was used as secondary antibody. Blots were visualized using the Pierce™ ECL Western Blotting Substrate kit (Thermo Fisher Scientific).

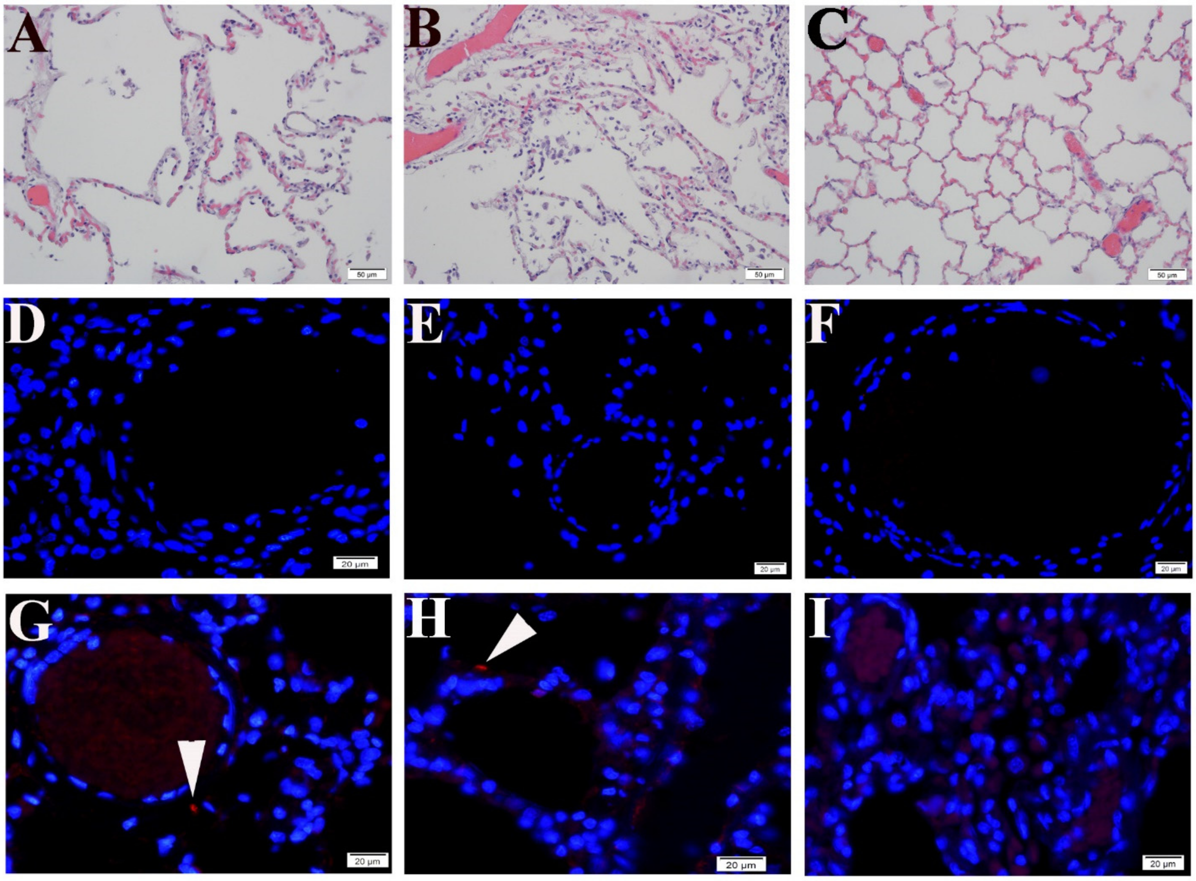

Figure S1. Different histological study controls. (**A-C**) H&E staining in normal human lung tissues from an archival paraffin tissue block. (**D**) Normal IgG controls in the IF assay. (**E**) Negative control from the TUNEL assay. (**F**) Negative control from the IF-TUNEL double labeling. IF staining of SARS-CoV-2 antigen (red) (arrow heads) in SARS-CoV-2 infected NHP lung tissues (**G** and **H**), compared with mock control (**I**).

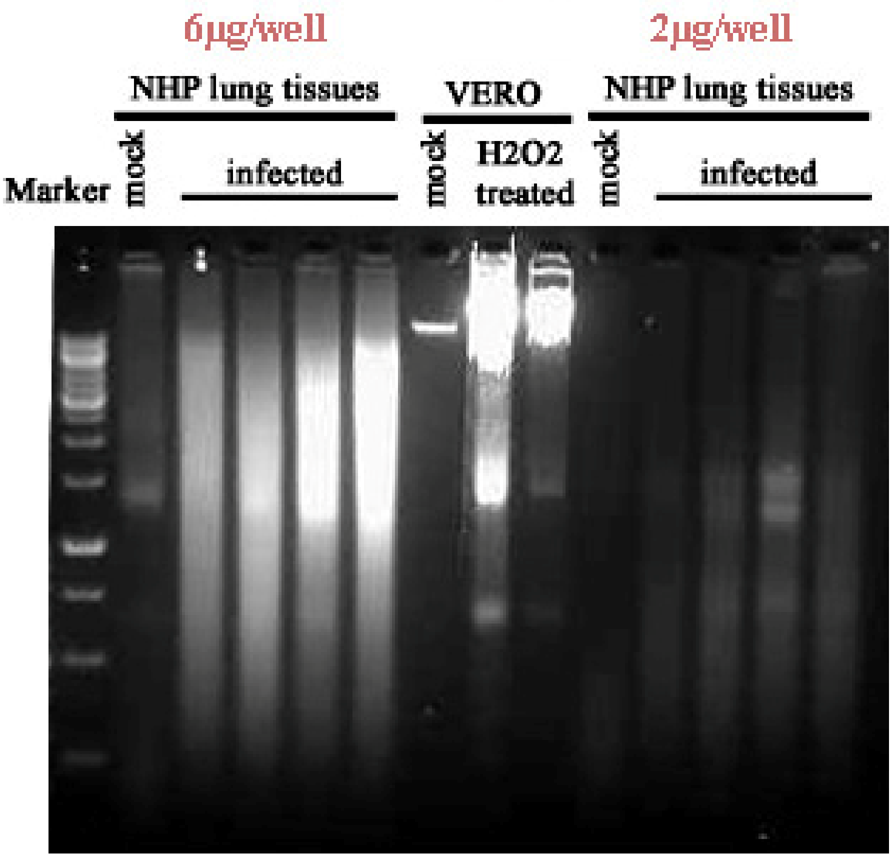

Figure S2. DNA fragmentation detected in SARS-CoV-2-infected NHP lung tissues. DNA fragmentations were detected in SARS-CoV-2 infected NHP lung tissue at different sample loading concentrations (2 μg vs. 6 μg), compared with mock control. H_2O_2 (500 μM for 48 hours)-treated Vero cells were used as a positive control.

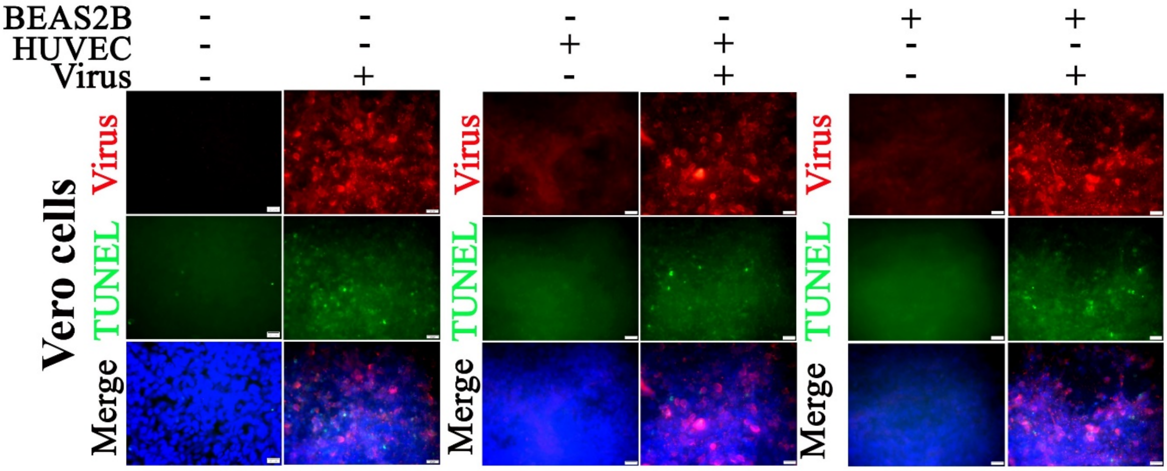

Figure S3. Apoptosis occurs in Vero cells when co-cultured with HUVECs or BEAS2B cells at 72 hours p.i. with SARS-CoV-2 infection. Fixed Vero cells in the insert were subjected to the procedures of IF assay to SARS-CoV-2 (red) and TUNEL assay (green). Nuclei of Vero cells were counterstained with DAPI (blue). Scale bars, 20 μm. .

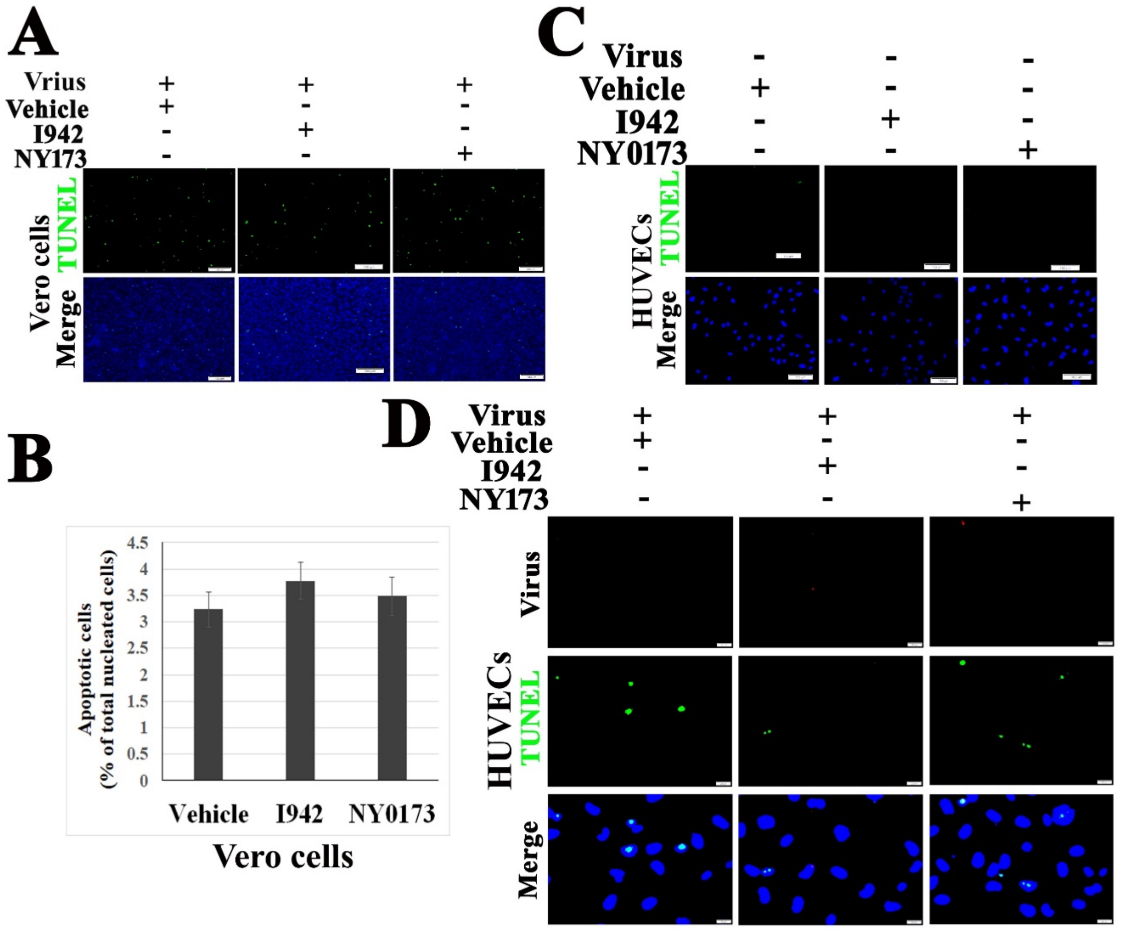

Figure S4. Pharmacological activator or inhibitor of EPAC induces no effect on apoptosis in Vero cells of the co-culture of Vero cells and HUVECs following SARS-CoV-2 infection. The co-cultures were exposed to 0.1 MOI of SARS-CoV-2 for 24 hours before treatment with NY0173 (5μM), I942 (5μM), and vehicle for 48 hours. (**A**) Fixed Vero cells in the insert were subjected to TUNEL assay (green). Nuclei of Vero cells were counterstained with DAPI (blue). Scale bars, 100 μm. (**B**) Percentage of TUNEL signal-positive Vero cells among all nucleated cells (DAPI staining) in each filed[5, 6]. The quantitative data presented are three independent experiments (n=18 in each group). *

compared to the vehicle group, *P* <0.01. (**C**) HUVECs of noninfected co-cultures were treated with NY0173 (5μM), I942 (5μM), and vehicle for 48 hours before TUNEL assay (green). Scale bars, 100 μm. (**D**) IF of viral antigens (red) and TUNEL (green) in HUVECs of the co-cultures exposed to 0.1 MOI of SARS-CoV-2 for 24 hours before treatment with NY0173 (5μM), I942 (5μM), and vehicle for 48 hours. Scale bars, 20 μm.

Supplemental Table S1: Clinical information and histopathological findings in post-mortem lung sections from COVID-19 patients.

Case	Gender	Age	Underlying diseases	H&E staining of lung tissues	TUNEL
#1	Male	43	Type 2 diabetes mellitus; hypertension.	Lymphocytic pneumonia; microthrombi, and lymphocytic endotheliitis; lymphocytic perivasculitis; widened septae with inflammatory cells	Positive
#2	Male	58	Hypertension	Lymphocytic pneumonia; microthrombi, and lymphocytic endotheliitis; lymphocytic perivasculitis; widened septae with inflammatory cells	Positive
#3	Male	54	Hypertension; urinary tract infection; hepatitis C; cirrhosis.	Lymphocytic pneumonia; microthrombi, and lymphocytic endotheliitis; lymphocytic perivasculitis; widened septae with	Positive

				inflammatory cells; intraalveola edema; early hyaline membrane formation	
#4	Male	57	Hypertension; type 2 diabetes mellitus; previous myocardial infarction.	Lymphocytic pneumonia; microthrombi, and lymphocytic endotheliitis; lymphocytic perivasculitis; widened septae with inflammatory cells; intraalveolar edema	Positive
#5	Male	68	Hypertension; cardiac failure; gout; gouty arthritis; urolithiasis; COPD.	Inflammatory cell infiltration; hyaline membrane formation; fibrin ball formation with fibrosis.	Negative

Note: The clinical course of patients, from first symptoms to death, ranged in length from 20 to 34 days with a mean of 27 days. SARS-CoV-2 RNA was detected in nasopharyngeal swab samples from all patients and was confirmed in post-mortem nasopharyngeal swab samples.

Supplemental Table S2: Relative frequency of cytochrome C- or FasL-positive apoptotic cells in NHP lung during SARS-CoV-2 infection (Percentage of the number of positive cells in one field under 20x fluorescent microscope of each case, mean± SEM).

	Uninfected control group (n=3)	Infected group (n=4)
Cytochrome C	0	(27.53 ± 3.38)%

FasL	0	(21.30 ± 3.35)%

Note: One field under fluorescent microscope (20x) was captured in one stained tissue section per case and image was processed with ImageJ[7] to calculated the ratio of both TUNEL- and cytochrome C- or FasL-positive cells to only TUNEL-positive cells.

Reference

1. Rockx B, Kuiken T, Herfst S, et al. Comparative pathogenesis of COVID-19, MERS, and SARS in a nonhuman primate model. *Science*. Apr 2020;doi:10.1126/science.abb7314
2. Munster VJ, Feldmann F, Williamson BN, et al. Respiratory disease in rhesus macaques inoculated with SARS-CoV-2. *Nature*. May 2020;doi:10.1038/s41586-020-2324-7
3. Liu Y, Xiao J, Zhang B, et al. Increased talin-vinculin spatial proximities in livers in response to spotted fever group rickettsial and Ebola virus infections. *Lab Invest*. Apr 2020;doi:10.1038/s41374-020-0420-9
4. He X, Drelich A, Yu S, et al. Exchange protein directly activated by cAMP plays a critical role in regulation of vascular fibrinolysis. *Life Sci*. Feb 2019;doi:10.1016/j.lfs.2019.02.014
5. Song Y, Coleman L, Shi J, et al. Inflammation, endothelial injury, and persistent pulmonary hypertension in heterozygous BMPR2-mutant mice. *Am J Physiol Heart Circ Physiol*. Aug 2008;295(2):H677-90. doi:10.1152/ajpheart.91519.2007
6. Daniel B, DeCoster MA. Quantification of sPLA2-induced early and late apoptosis changes in neuronal cell cultures using combined TUNEL and DAPI staining. *Brain Res Brain Res Protoc*. Aug 2004;13(3):144-50. doi:10.1016/j.brainresprot.2004.04.001
7. O'Brien J, Hayder H, Peng C. Automated Quantification and Analysis of Cell Counting Procedures Using ImageJ Plugins. *J Vis Exp*. 11 2016;(117)doi:10.3791/54719
